# Anti-depressant and anxiolytic potential of *Acacia hydaspica* R. Parker aerial parts extract: Modulation of brain antioxidant enzyme status

**DOI:** 10.1186/s12906-017-1671-x

**Published:** 2017-04-24

**Authors:** Tayyaba Afsar, Suhail Razak, Muhammad Rashid Khan, Ali Almajwal

**Affiliations:** 1 Department of Biochemistry, Faculty of Biological Sciences, Quaid-i-Azam University, Islamabad, Pakistan; 2 Department of Animal sciences, Faculty of Biological Sciences, Quaid-i-Azam University, Islamabad, Pakistan; 3 Department of Community Health Sciences, College of Applied Medical Sciences, King Saud University, Riyadh, Saudi Arabia

**Keywords:** Depression, Force swimming test, Immobility time, GCMS, Brain antioxidants, Phytochemicals

## Abstract

**Background:**

Oxidative stress may link to psychiatric disorders, and is being regarded as a plausible mechanism that can affect the regulation of these illnesses. The present study was undertaken to investigate the antidepressant and anxiolytic potential of *A. hydaspica* R. parkers. Brain oxidative stress enzyme levels were analyzed to correlate depression and stress with brain antioxidant status.

**Methods:**

Antidepressant and anxiolytic effect of methanol extract of *A. hydaspica* and its derived soluble fractions [*n*-hexane (AHH), ethyl-acetate (AHE), chloroform (AHC), *n*-butanol (AHB) and remaining aqueous fraction (AHA)] was investigated by using three behavioral models; the Forced swimming test, Tail suspension test and Elevated plus-maze test (EPM). Chronic unpredictable mild stress (CMS) was employed to induce stress in rats. AHM and AHE (200 mg/kg, *p.o*), fluoxetine (5 mg/kg, *i.p*) and diazepam (DZM) (1 mg/kg, *p.o*) were administered during the 7 day stress exposure period, and rats were assessed for antidepressant and anxiolytic behavioral despair paradigms. Antioxidant enzymes and oxidative stress markers were measured in brain tissue of depressed rats. Phytochemical analysis was done by GCMS experimentation.

**Results:**

AHM and AHE (acute dose) significantly (*p* < 0.0001) reduced the immobility time and ameliorated climbing behavior as compared to the control in FST and TST, and similar to fluoxetine. AHM and AHE showed significant (*p* < 0.0001) anxiolytic potential in EPM, and comparable to DZM (1 mg/kg b.w., *i.p*). Significant decrease in antioxidant enzyme levels and increase in MDA, H_2_O_2_ and NO level were observed in stressed rats. AHM and AHE (for 7 days/CMS) significantly improved behavior in FST, TST and EPMT. Treatment also improved antioxidant enzyme level and controlled the oxidative stress markers in brain tissues. GCMS analysis indicated the presence of 10 different chemical constituents in *A. hydaspica*.

**Conclusion:**

The present study revealed that *A. hydaspica* exerts an antidepressant and anxiolytic effect by improving brain antioxidant status. The observed activities might be due to the presence of diverse phytochemicals.

**Electronic supplementary material:**

The online version of this article (doi:10.1186/s12906-017-1671-x) contains supplementary material, which is available to authorized users.

## Background

Depression is the most prevalent enduring complaint in clinical observations [1] and will turn out to be the 2nd dominant cause of premature deaths or developmental disabilities globally by the year 2020 [2]. Even though a large percentage of depressed patients responded to the presently employed treatments [3], but the extent of improvement is still disappointing, coupled with the various physiological side effects and tolerance on chronic treatment. Stress play the main role in pathogenesis of mental disorders [4, 5]. Stressful conditions can precipitate anxiety and depression, which leads to excessive production of free radicals which in turn results in oxidative stress [6]. Also abnormal oxidative product levels were seen in the peripheral blood [7], red blood cells (RBC) [8], urine [9], cerebrospinal fluid and postmortem brains of depressed patients [10]. Brain has intrinsic oxidative vulnerability. When the production of ROS prevails over the brain defense systems, the lipid-rich constitution of brain might favor the lipid peroxidation in conjunction with defective antioxidant defenses, constituting a free radical chain reaction that may alter overall brain activities [11]. Tripeptide glutathione (GSH), a redox regulator, participates in the maintenance of oxidant homeostasis and the cellular detoxification of ROS in brain cells. GSH depletion has been shown to affect mitochondrial function probably via selective inhibition of mitochondrial complex I activity. Therefore, compromised GSH system in the brain has been considered as a relevant index of neuronal oxidative stress [12]**.** Currently different therapeutic regimens are employed to treat anxiety and depressive disorders; but their clinical uses are limited by their side effects. In current scenario, we need drugs with lesser side effects and maximum efficacy. Hence Ayurveda has recently become the drug of choice. Globally herbal medicines are extensively used due to their therapeutic efficiency and minimum side effects in neurological disorders, therefore investigations for the search of novel and better tolerated molecules from plant sources have progressed constantly, demonstrating the pharmacological effectiveness of different plant species in a variety of animal models [13].

*Acacia hydaspica* R.Parker, family Leguminosae is an ethnobotanically important plant. The bark and seeds are source of tannins. Gallic acid, catechin, rutin and caffeic acid have been identified in *A. hydaspica* by HPLC-DAD screening [14], 7-*O*-galloyl catechin, + catechin and methyl gallate have been isolated from ethyl acetate fraction of *A. hydaspica* (AHE) [15]. Pharmacological studies carried out with extracts from *A. hydaspica* showed that it possess several biological effects, such as: anti-inflammatory, analgesic, antipyretic, cytotoxic, antioxidant, anti-hemolytic and anticancer potential due to the presence of active secondary metabolites [14–16].

Various species from the family Leguminosea were reported to possess anti-depressant activity for example; intravenous administration of lectins obtained from the seeds of *Canavalia brasiliensis* considerably decline immobility interval of male Swiss albino mice in force swimming tests (FST). Furocoumarins extracted from the seeds of *Psoralea corylifolia* L. substantially lessen the immobility interval in FST in male mice and higher doses showed more significant effects compared to amitryptyline (10 and 20 mg/kg) and fluoxetine (13 mg/kg) [17]. Chhillar and Dhingra, 2013 illustrated the antidepressant like activity of gallic acid in anxious and un-anxious mice, probably due to its antioxidant potential and via inhibition of MAO-A action, fall in plasma nitrite and corticosterone levels. Rutin, a flavonoid possess antidepressant activity [18]. The identification of rutin, gallic acid, catechin, and other polyphenols from *A. hydaspica* prompted us to assess the anti-depressant potential of *A. hydaspica*.

However to the best of our knowledge there is no single scientific report demonstrating the anti-depressant and anxiolytic activity of *A. hydaspica*. Current investigation aimed at evaluating the antidepressant and anxiolytic potential of *A. hydaspica*, furthermore the brain antioxidant enzymes were determined and chemical profiling of crude methanol extract was done by GCMS analysis.

## Methods

### Plant collection

The aerial parts (bark, twigs, and leaves) of *A*. *hydaspica* were collected and their specimen (Accession No. 0642531) was deposited at the Herbarium of Pakistan, Museum of Natural History, Islamabad. They were shade dried at room temperature, chopped and ground mechanically to a mesh size of 1 mm.

### Preparation of extract/fractions

**Fig. 1:**
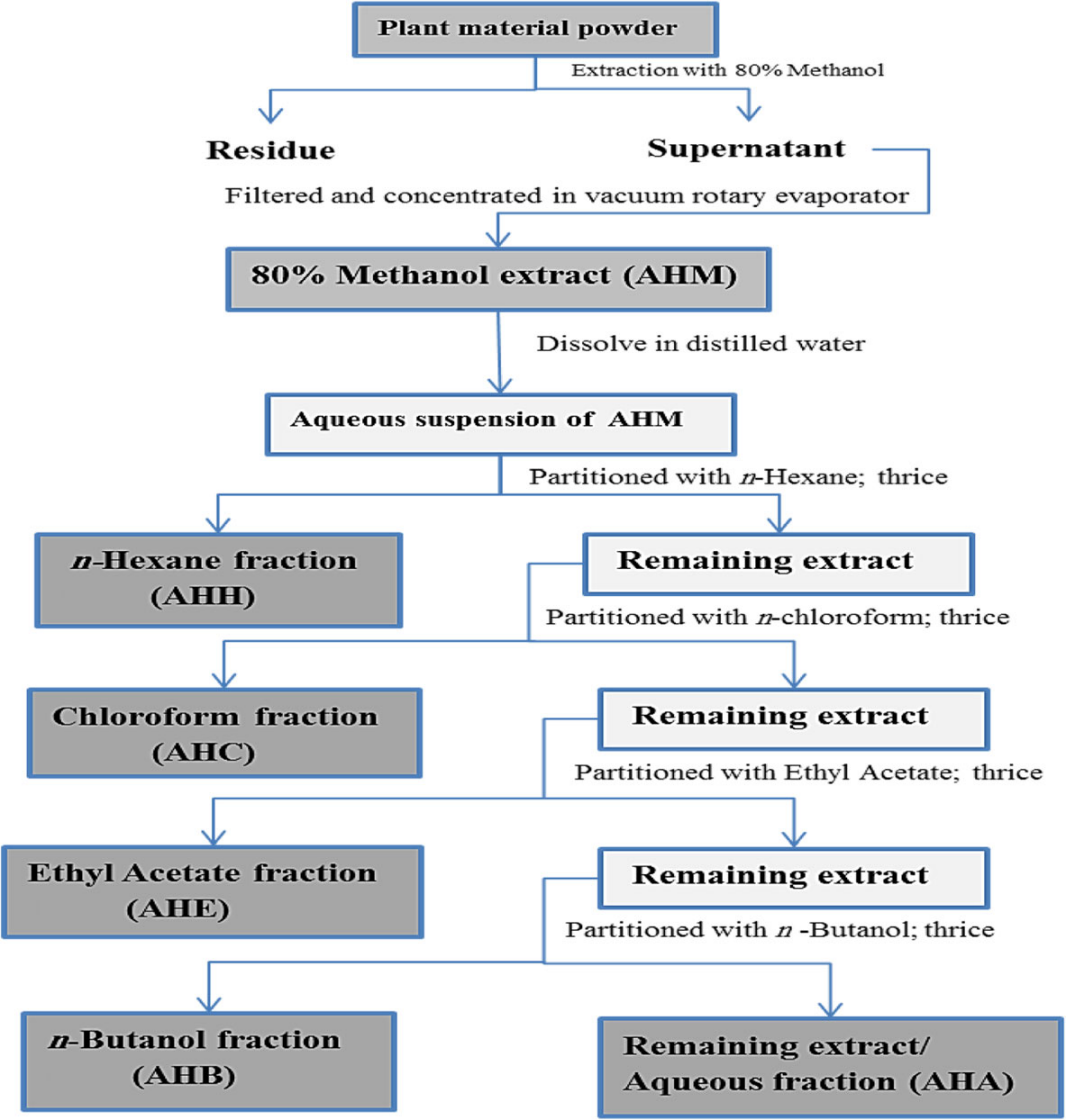
Flow chart describing the extraction procedure for fractionation of *A. hydaspica*

3 kg plant powder was macerated in methanol (3 × 4000 ml) for five consecutive days. The supernatant was filtered and concentrated by rotary evaporator (Buchi, R114, Switzerland) under reduce pressure at 40 °C to obtain crude methanol extract (AHM). AHM (12 g) was dissolved in water (250 ml) and successively fractionated (3 × 200 ml) with following solvents; *n*-hexane (AHH), ethyl acetate (AHE), chloroform (AHC) and *n*-butanol (AHB) respectively, and layers were allowed to isolate for 3 h in a separating funnel, and finally residual aqueous fraction was obtained (AHA). All fractions were dried using a rotary evaporator and stored at 4 °C for subsequent investigation. The scheme of extraction/fractionation is described in Fig. 1.

### Phytochemical screening

#### Gas chromatography-mass spectroscopy (GC-MS) analysis

GC-MS screening of methanol extracts of *A. hydaspica* was done on Thermo GC -Trace Ultra version 5.0, equipment coupled with Thermo MS DSQ II mass spectrometer. Compound mixture was separated on a ZB 5-MS capillary standard non-polar column (30 m × 0.25 mm ID × 0.25 μm FILM). The column temperature was set at 70 °C for 2 min, 70–260 °C at 6 °C/min, and as a final point held for 10 min at 260 °C. The particle-free diluted crude methanol extract sample was injected in split-less mode (split flow: 10 ml/min, split less time 1 min). Carrier gas (helium) was employed at a constant flow rate of 1 ml/min and 1 μl sample was injected. Relative percentages of crude extract constituents were expressed as peak area normalization. Mass spectral scan range was set at full scan mode from 50 to 650 (m/z) [19, 20]. Total GC running time was 37.52 min. The identification of unknown chemical constituents in extract was based on comparison of their retention indices and mass spectra fragmentation patterns with those stored on the Wiley9 and mainlab computer library search program; built up using pure substances or with authentic compounds [21]. The GC spectra, MS fragmentation pattern of each peak at different retention times (RT) and their library matches were provided in Additional file 1 (GCMS fragmentation pattern and compounds structure).

### Animals

Fourty-eight, adult male Sprague Dawley rats (180–190 g, Primate Facility, Quaid-i-Azam University, Islamabad) were used for acute dose testing in the FST, TST and EPM. 30 adult male Sprague Dawley rats (180–190 g) were used for 1 week dose experiment in the FST, TST, EPM and brain antioxidant enzyme testing.

Animals were kept in ordinary cages at room temperature of 25 ± 3 °C with a 12 h dark/light cycle. They were allowed free access to food in form of dry pellets and water and randomly divided into different experimental groups. The study protocol was approved by Ethical committee of Quaid-i-Azam University Islamabad for laboratory animal feed and care.

### Drugs and treatments

All fractions were tested for initial screening of antidepressant and anxiolytic potential. Extract/fractions with potential antidepressant and anxiolytic potential were further tested for evaluated to link antioxidant activity with depression and anxiolytic potential.

### Acute dose experiment

#### Antidepressant model

##### Forced swim test (FST)

The FST is a pharmacological method for estimating antidepressant potential in animal models [22]. The apparatus comprised of a clear plexiglass cylinder (30 cm high and 12 cm diameter) containing 25 cm of water. 24 h prior to swimming test, animal were assessed in the pretest and placed individually for 15 min in the cylinder. All the animals were dried properly before placing them back into their own cages. All the rats were divided in to three groups (*n* = 6).

Group I: Control group received normal saline (5 ml/kg, i.p), Group II: Standard group received fluoxetine (10 mg/kg b.w, i.p), 1 h before swimming test, Group III: Represented as plant treated group, which was further divided in to 6 groups i.e.; III a, III b, III c, III d, III e and III f, received 200 mg/kg, oral dose of AHM, AHH, AHE, AHC, AHB and AHA respectively.

In total time period of 8 min, the duration of immobility was recorded during the last 5 min [23]. After an initial 3 min period of vigorous activity each animal assumed a typical immobile posture. A rat was considered to be immobile when it remained floating motionless in the water, ceased for struggling and making only minimum movements of its limbs necessary to keep its head above water. Changes in duration of immobility of each group were studied in this model.

##### Tail suspension test (TST)

The tail suspension test was performed according to previously established procedure on rats [24]. Before the initiation of behavioral experiment rats were permitted to acclimatize for 24 h at room temperature. 1 h after administration of different treatments; rats were separately suspended by the tail from horizontal bar (75 cm above the table top) by using tape. Duration of immobility was noted by a trained observer for 5 min.

#### Anxiety model

##### Elevated plus-maze test

Behavior in the elevated plus-maze (EPM) [25], is utilized to assess exploration, anxiety, and motor behavior. The EPM consists of four arms, 49 cm long and 10 cm wide, elevated 50 cm above the ground. Two arms were enclosed by walls 30 cm high and the other two arms were exposed. 30 min after the administration of the extract/fractions, each rat was placed in the center of the maze facing one closed arm. Behavior was observed for 5 min., and the time spent and number of entries into the open and enclosed arms was counted. The percentages of time spent in the open arms (time spent in the open arms/time spent in all arms × 100) were calculated. In addition, the total number of open- and enclosed-arm entries (number of crossing), which indicates the exploratory activity of animals, was measured. An entry was defined as an animal placing all four paws into an arm, and no time was recorded when the animal was in the central area. The maze floor was cleaned with cotton and 10% ethanol solution between subjects.

### Repeat dose experiment

#### Induction of stress

Stress was induced by employing the Chronic Unpredictable Mild Stress (CMS) protocol with slight modifications [6]. Each stress regimen was carried out for 1 period with the following stressors: food deprivation for 24 h, cage tilting (~45 degree inclined) for 22 h, crowded housing (10 animals per cage) for 12 h, cold stress 4–8 °C and heat stress 38–39 °C for 20 min.

#### Experimental grouping

The extract/fractions which exhibit significant activity in first trial of experiments were selected for repeat dose experiment. The experiment was designed following the protocol of [25, 6] with modifications. Animals were exposed to CMS and grouped as follow: Group I: Control (saline 200 μl, i.p.), Group II: AHM-treated group (*n* = 6, 200 mg/kg b. w., *p.o*), Group III: AHE-treated group (*n* = 6, 200 mg/kg b.w., *p.o*), Group IV: Fluoxetine treated group (*n* = 6, 5 mg/kg, b. w., *i.p*.), and Group V: Diazepam-treated group (*n* = 6, 1 mg/kg b. w, *i.p*.). All these doses were given to rats for one week. The rats were sacrificed on the 8th day after FST, TST and EPM experiments and, various antioxidants and oxidative stress markers enzymes were estimated.

#### Preparation of brain homogenate

After completion of experiments rats were killed and, whole intact brain was carefully removed and rinsed with 0.9%Nacl solution for cleaning, and weighed. Brain tissue was homogenized in a phosphate buffer (pH 7.6), centrifuged at 20,000×g, 4 °C for 10 min. An aliquot of supernatant was collected and stored at −20 °C for furthers biochemical tests. Protein concentrations were determined by the Bradford assay with Bovine serum albumin as standard (0.05–1.00 mg/ml).

#### Assessment of brain antioxidant markers

##### Catalase (CAT) activity

CAT activity was determined by the protocol of Khan et al. with slight modifications [26]. The CAT reaction solution consists of 625 μl of 50 mM of potassium phosphate buffer (pH 5), 100 μl of 5.9 mM H_2_O_2_ and 35 μl enzyme extract. Change in the absorbance of the reaction solution was noted after 1 min at 240 nm. An absorbance change of 0.01 as units/min denotes one unit of catalase activity.

##### Superoxide dismutase (SOD) activity

Kakkar et al. method was utilized for the assessment of SOD activity [27]. Phenazine methosulphate and sodium pyrophosphate buffers were exploited for the assessment of SOD activity. Centrifugation of tissue homogenate was done at 1500×g for 10 min and then at 10,000×g for 15 min. Supernatant was collected and 150 μl of it was added to the aliquot containing 600 μl of 0.052 mM sodium pyrophosphate buffer (pH 7.0) and 186 mM of phenazine methosulphate (50 μl). 100 μl of 780 μM NADH was added to initiate enzymatic reaction. After 1 min, glacial acetic acid (500 μl) was added to stop the reaction. At 560 nm optical density was determined to enumerate the color intensity. Results were evaluated in units/mg protein.

##### Glutathione-S-transferase assay (GST)

Scheme of Habig et al. [28] was followed for the estimation of GST potency. 150 μl aliquot of tissue homogenate was added to 720 μl of sodium phosphate buffer together with 100 μl of reduced glutathione (1 mM) and 12.5 μl of CDNB (1 mM). The changes in the absorbance were recorded at 340 nm and enzymes activity was calculated as nmol CDNB conjugate formed/ min/mg protein using a molar extinction coefficient of 9.6 × 103 M-^1^ cm-^1^.

##### Glutathione reductase assay (GSR)

Glutathione reductase activity in tissue samples was analyzed as described by Carlberg and Mannervik [29]. The reaction reagent 2 ml was made of of 1.65 ml phosphate buffer: (0.1 M; pH 7.6), 100 μl EDTA (0.5 mM), 50 μl oxidized glutathione (1 mM), 100 μl NADPH (0.1 mM) and 100 μl of homogenate. Activity of enzyme was monitered by recording the absorbance of the vanishing of NADPH at 340 nm at 25 °C. Estimated of enzyme level was accomplished as nM NADPH oxidized/min/mg protein by employing molar extinction coefficient of 6.22 × 10^3^/M/cm.

##### Glutathione peroxidase assay (GPx)

Glutathione peroxidase activity was assessed as described elsewhere [30]. Entire volume of 2 ml reaction solution comprised of 1 mM EDTA (100 μl), 0.1 M phosphate buffer (1.49 ml; pH 7.4), 1 m M sodium azide (100 μl), 1 IU/ml glutathione reductase (50 μl), 1 mM GSH (50 μl), 0.2 mM NADPH (100 μl), 0.25 mM H_2_O_2_ (10 μl) and tissue homogenate (100 μl). The loss of NADPH was recorded at 340 nm at room temperature. Enzyme level was estimated as nM NADPH oxidized/min/mg protein employing 6.22 × 10^3^/M/cm molar extinction coefficient.

##### Reduced glutathione assay (GSH)

Reduced glutathione activity was checked as described by Jollow [31], using DTNB as a substrate. The yellow color developed was read immediately at 412 nm and expressed as μmol GSH/g tissue.

#### Assessment of oxidative stress markers

##### Estimation of a MDA

Malondialdehyde (MDA) is one of lipid peroxidation product that can be used as a marker of oxidative stress. The assay was carried out following the previous protocol [32]. The reaction mixture in a total volume of 1.0 ml contained 0.58 ml phosphate buffer (0.1 mol; pH 7.4), 0.2 ml homogenate sample, 0.2 ml ascorbic acid (100 mmol), and 0.02 ml ferric chloride (100 mmol). The reaction mixture was incubated at 37 °C in a shaking water bath for 1 h. The reaction was stopped by addition of 1.0 ml 10% trichloroacetic acid. Following addition of 1.0 ml 0.67% thiobarbituric acid, all the tubes were placed in boiling water bath for 20 min and then shifted to ice bath before centrifuging at 2500×g for 10 min. The amount of TBARS formed in each of the samples was assessed by measuring optical density of the supernatant at 535 nm using a spectrophotometer against a reagent blank. The results were expressed as nmol TBARS/min/ mg tissue at 37 °C using a molar extinction coefficient of 1.56 × 105 M-^1^ cm-^1^.

##### Hydrogen peroxide assay

In the reaction mixture, 500 μl of 0.05 M phosphate buffer (pH 7), 100 μl of homogenate was added along with 100 μl of 0.28 nM phenol red solution, 250 μl of 5.5 nM dextrose and horse radish peroxidase (8.5 units) was added. Incubation was done at room temperature for 60 min. 100 μl of NaOH (10 N) was added to stop the reaction. After centrifugation for 5–10 min at 800×g, absorbance of the supernatant was calculated at 610 nm. Production of H_2_O_2_ was measured as nM H_2_O_2_/min/mg tissue by employing the standard curve of phenol red oxidized by H_2_O_2_ [33].

##### Nitrite assay

Briefly, tissue samples (100 mg each) were de-proteinised in 100 μl solution comprising 5% ZnSO_4_ and 0.3 M NaOH. Samples were Centrifuge at 6400×g for 15–20 min. Remove supernatant and add 20 μl in a cuvette containing 1 ml of Griess reagent, change in color was determined at 540 nm. Griess reagent 1 ml was used as a blank in the spectrophotometer (Smart Spec TM Spectrophotometer). Standard curve of sodium nitrite was utilized for the quantification nitrite concentration in testicular tissues [34].

## Results

### GC-MS analysis of *A. hydaspica*

**Fig. 2:**
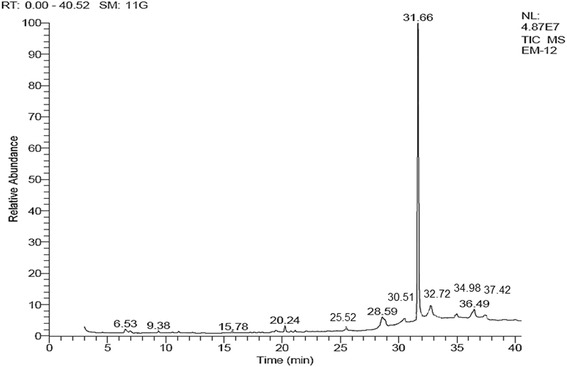
GC Chromatogram of *A. hydaspica* crude methanol extract (AHM)

Compound composition of *A. hydaspica* crude extract (AHM) was investigated by GC-MS analysis. This technique was employed to get the fingerprint of plant secondary metabolites (volatile compounds). The compounds were identified through mass spectrometry attached with GC. The mass spectrometer analyzes the compounds eluted at different times to identify the nature and structure of the compounds. The large compound fragments into small compounds giving rise to appearance of peaks at different m/z ratios. These mass spectra are fingerprint of that compound which can be identified from the data library. The chromatogram reveals the presence of various chemical components with different retention times as illustrated in (Fig. 2). The main chemical constituents identified in the plant extract according to their peak area percentages are 1,2-Benzenedicarboxylic acid mono (2-ethylhexyl) ester (70.65%), α-Amyrin (5.03%), Vitamin E (4.56%), 2,6-dimethyl-N-(2-methyl-à-phenylbenzyl) aniline (2.51%) and Squalene (4%). Other compounds with less peak area (%) were shown in Table 1. The fragmentation pattern of different compound peaks, their database search matches on the basis of probabibily and compounds structure were presented in Additional file 1 (GCMS fragmentation pattern and compounds structure). The composition determined for this methanol extract corresponds to 93.62% of the entire GC-MS chromatogram. The present study helps to predict the formula and structure of 10 biomolecules. Further investigation may lead to isolation of bioactive compounds and structural elucidation.

**Table 1:** Chemical composition of *A. hydaspica* AHM extract analyzed by GCMS *P* Peak, *RT* Retension time, *SI* Search index, *RSI* Reverse search index

P#	RT	Compound Name	SI	RSI	Mol. formula	Mol. weight	% Peak Area	Nature
1	6.53	2-(4'-Nitro-3'-Thienyl)Pyrimidine	377	623	C_8_H_5_N_3_O_2_S	207	1.62	Heterocycle containing Nitrogen
2	20.24	Phytol acetate	871	879	C_22_H_42_O_2_	338	1.28	Diterpene
3	25.52	2-Hexadecen-1-ol,3,7,11,15-tetramethyl-, [R-[R*,R*-(E)]]- (CAS), Phytol (CAS)	701	801	C_20_H_40_O	296	1.16	Alcohol
4	28.59	α-Amyrin	660	972	C_30_H_50_O	426	5.03	Triterpene
5	30.51	N,N'-Dicyclohexyl-1-cyano-7-pyrrolidinyl perylene-3,4:9,10-tetracarboxylic acid Bisimide	883	883	C_41_H_36_N_4_O_4_	648	1.80	Polycyclic aromatic hydrocarbon
6	31.66	1,2-Benzenedicarboxylic acid, mono (2-thylhexyl) ester	931	931	C_16_H_22_O_4_	278	70.65	ester
7	32.75	Vitamin E	762	913	C_29_H_50_O_2_	430	4.54	Vitamin
8	34.98	2,6-dimethyl-N-(2-methyl-à-phenylbenzyl) aniline	506	550	C22H23N	301	2.51	Aniline, aromatic amine
9	36.49	Squalene	491	525	C30H50	410	4.00	Triterpene
10	37.42	Cyclohexane,1,1,3-trimethyl-2,3-epoxy-2-(3-methylcyclobuten-2-yl-1)-4-acetyloxy-/1,5,5-Trimethyl-6-(3-methyl-2-cyclobuten-1-yl)-7-oxabicyclo[4.1.0]hept-2-yl acetate	414	485	C16H24O3	264	1.03	Oxirane

### Antidepressant activity

#### Effect of a. Hydaspica on forced swim test (FST)

**Fig. 3:**
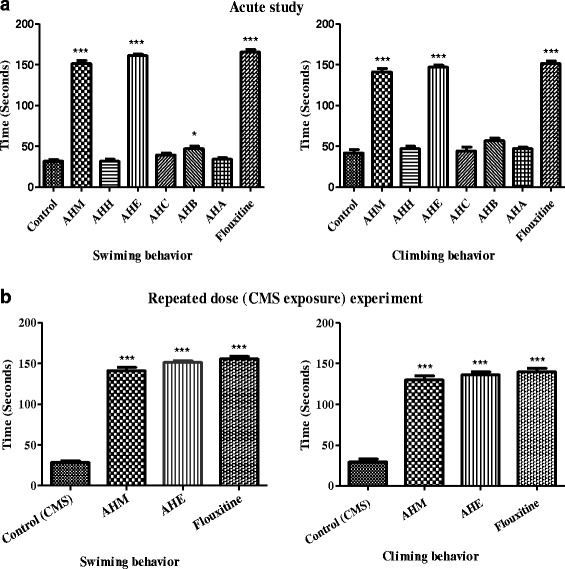
Effect of acute and repeated dose of *A. hydaspica* extract/fraction (200 mg/kg b.w, *p.o*) and fluoxetine (10 mg/kg b.w, *i.p*.) on behavioral pattern in forced swing test in rats. **a** Effect acute dose on swimming and climbing behavior, **b** Effect repeated doses of AHM, AHE and fluoxetine on swimming and climbing behavior. Data presented as mean ± SEM (*n* = 6). Asterisks ***, * indicate significant (*p* < 0.001 and *p* < 0.05 respectively) difference from respective control. Data analyzed by one way ANOVA followed by Tukey’s multiple comparison tests

Effect of various treatments on immobility behavior in FST in both acute and repeated doses (CMS exposure) experiments is shown in Table 2. AHM and AHE markedly (*p* < 0.001) decreased the immobility time in comparison to respective control group while non-significant difference in activity was noticed in contrast to fluoxetine (Table 2). The effect of various treatments in acute and repeated doses (CMS exposure) on swimming period is shown in Fig. 3a. AHM and AHE significantly increased the swimming period in both acute and repeated dose experiments. AHE exhibited analogous activity to fluoxetine. Climbing period was also enhanced markedly (*p* < 0.001) by the AHM and AHE (Fig. 3b) in both experiments. Maximum increase in climbing time was observed with fluoxetine followed by AHE and AHM. Climbing period of AHE and fluoxetine was statistically similar (*p* > 0.05).

**Table 2:** Antidepressant like effect of *A. hydaspica* in acute and repeated doses in FST and TST (Immobility behavior) models

Treatment	FST (Immobility time in seconds)	TST (Immobility time in seconds)
*Acute dose study*		
Control	171.0±3.79	192.3±2.85
AHM	33.33±2.85***	37±2.52***
AHH	168.0±3.51b	181.3±1.86
AHE	32.0±2.52***	35.67±2.73***
AHC	166.7±2.33b	183.3±1.45
AHB	156.0±3.79*	179.3±2.33*
AHA	172.0±2.52	182.0±3.60
Fluoxetine	30.33±2.03***	34.67±2.91***
*1 week dose study*		
Control (CMS)	189.0±2.79	204.3±3.01
AHM	35.36±2.55***	38.2±2.32***
AHE	34.51±3.01***	35.67±2.63***
Fluoxetine	33.13±2.63***	36.77±2.98***

Data presented as mean±SEM (*n*=6). Asterisks *, *** indicate significant (*p*<0.05 and *p*<0.0001 respectively) difference from respective control groups in both experiments. Data analyzed by one way ANOVA followed by Tukey’s multiple comparison tests

#### Effect of *A. hydaspica* on tail suspension test (TST)

Table 2 displayed significant variations in the results of different treatment on immobility behavior in TST in both acute and repeated dose experiments. The change in immobility actions induced with AHM and AHE were statistically similar to fluoxetine.

### Anxiolytic activity

#### Effect of *A. hydaspica* extract/fraction on elevated plus-maze behavior

**Fig. 4:**
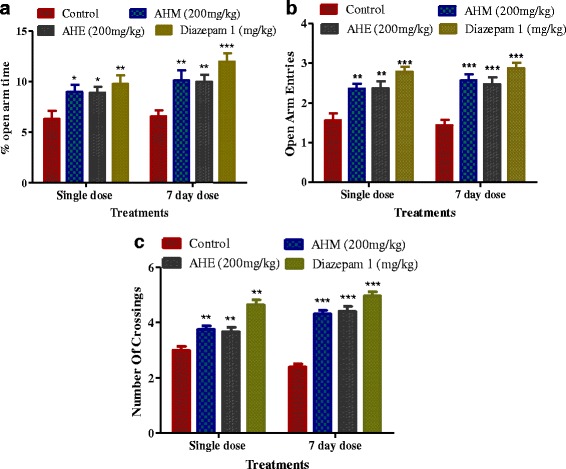
Effects of the acute and repeated dose of AHM and AHE in the elevated plus-maze test on the percentage of the time spent in the open arms (**a**), the number of open-arm entries (**b**) and the number of crossing (**c**) in depressed rats. Values are means + SEM (*n* = 6). Data analyzed by one way ANOVA followed by Tukey’s multiple comparison tests

The elevated plus-maze task (Fig. 4), revealed a significant overall differences between all groups on the percentage of the time spent in the open arms. AHM and its fraction AHE showed significant effect in acute dose study compared with other fractions, especially AHE, more considerably increased the percentage of the time spent in the open arms as compared to control group. Additionally, repeated dose analysis of AHM and AHE revealed a more significant difference compared to control group, while nonsignificant differences were noted compared to diazepam treated group.

### Animal body weight and brain weight

No significant changes were observed in body weight of treated and control rats after seven days. Similarly, no considerable differences in the wet weight of the whole brain between control, AHM and AHE treated rats (Table 3).

**Table 3:** Body weight before and after treatment and wet brain weight

Treatment	Control (CMS)	AHM (200mg/kg)	AHE (200mg/kg)
Initial body weight (g)	185.2 ± 2.9	186.6 ± 3.2	186.9 ± 4.1
Final body weight (g)	199.4 ± 4.4	196.5 ± 3.8	197.8 ± 5.2
Wet brain weight (mg)	361.5 ± 3.4	363.1 ± 4.1	360.4 ± 3.4

Mean ± S.E.M values (*n* = 6/group). BT

### Effect of *A. hydaspica* on brain oxidative status in stressed rats

Application of different stressors for 7 days and behavior despair tests significantly depleted antioxidant enzymes activity in brain tissue of CMS (control) rats. AHM and AHE (200 mg/kg) significantly (*p* < 0.0001) improved; CAT, SOD, GSH, GR, GST, γ-GT and GPx enzymes activity in brain tissue. The effects of plant treatments were similar to standard drugs (Table 4). Depression and anxiety significantly decrease brain tissue protein content and increase the oxidative stress markers viz., lipid peroxidation (MDA content), H_2_O_2_ and nitrite contents in control untreated group (Table 5). AHM and AHE treatments significantly (*p* < 0.0001) augment the suppressed protein content while decreasing the oxidative stress markers in contrast to control (CMS) group.

**Table 4:** Effect of oral dose of AHM and AHE for 7 days on brain antioxidant status

Group	SOD (U/mg protein)	CAT (U/min)	GR (nM/min/mg protein)	GST (nM/min/mg protein)	GSH (μM/g tissue)	GPx (nM/min/mg Protein)
Control (CMS)	0.79±0.027	9.035±0.09	108.7±1.09	108.9±1.105	12.36±0.490	63.2±1.027
AHM	1.15±0.037***	15.76±0.118***	154.9±0.96***	148.6±0.665***	20.55±0.280***	122.4±0.639***
AHE	1.14±0.049***	15.66±0.197***	155.6±0.439***	148.4±0.815***	20.66±0.237***	122.9±0.285***
Diazepam	1.04±0.025**	14.95±0.216***	141.2±1.00***	140.6±0.723***	18.59±0.44***	114.1±0.845***
Fluoxetine	1.06±0.053**	14.93±0.256***	140.3±1.55***	139.2±0.4623***	18.77±0.360***	115.9±1.29***

Values expressed as mean±SEM (*n*=6). **, ***: Significant difference at *p* < 0.001 and *p* < 0.0001 *vs*. control group respectively. (One way ANOVA followed by Tukey’s multiple comparison tests)

**Table 5:** Effect of oral dose of AHM and AHE for 7 days on brain tissue protein and oxidative stress markers

Group	Protein (μg/mg Tissue)	H_2_O_2_ (nM/min/mg Tissue)	Nitrite (content μM/ml)	TBARS (nM/min/mg protein)
Control (CMS)	1.25±0.032	5.854±0.011	78.12±0.499	7.575±0.573
AHM	1.372±0.033*	1.932±0.015***	42.59±0.552***	2.874±0.180***
AHE	1.367±0.015*	1.951±0.049***	41.72±0.650***	2.859±0.086***
Diazepam	1.363±0.018*	2.119±0.006***	49.94±0.770***	3.258±0.167***
Fluoxetine	1.37±0.027*	2.645±0.004***	50.60±0.32***	3.233±0.151***

Values expressed as mean±SEM (*n*=6). *, ***: Significant difference at *p* < 0.05 and *p* < 0.0001 *vs*. control group respectively. (One way ANOVA followed by Tukey’s multiple comparison tests)

## Discussion

Depression and anxiety, represents one of the major health problems among other mood disorders worldwide. The execution of suitable animal models is essential for understanding of the neurobiological basis of mood disorders and is facilitating the approaches for the discovery of novel therapeutic targets. Forced Swim Test (FST) and Tail Suspension Test (TST) are employed as an exemplary systems to probe depressing condition in rodents [35], whereas elevated plus maze test (EPM) is a widely used behavioral assay for rodents and it has been validated to assess the anti-anxiety effects of pharmacological agents [36]. Immobility or despair behavior produced in both FST and TST were hypothesized to display animal’s hopelessness and low mood (behavioral despair), and are taken as paradigm of depression. This simple behavioral procedure is widely used test for screening novel antidepressants [37].

The result of current investigation exposed the significant antidepressant and anxiolytic potential of AHM and its fraction AHE as compared to other derived fractions. Recorded immobility period with AHM and AHE was noticeably short as compared to that of control in both acute and repeated dose experiments. On the other hand, immobility timing of AHM and AHE were similar to that of fluoxetine (standard antidepressant drug); which is a serotonin reuptake inhibitor, anticipated similar mechanism of action of AHM, AHE and fluoxetine [38]. The reduction in immobility timing caused by fluoxetine was accompanied by an increase in swimming and climbing behavior. Previous reports indicated that polyphenolic and poly-herbal formulation showed comparable antidepressant activity with that of standard antidepressant by increasing the level of noradrenaline and serotonergic transmission in brain of antidepressant models [39]. Our speculation is that *A. hydaspica* plant might possess antidepressant and anxiolytic activity owing to the presence of polyphenolic compounds and antioxidant potential [40]. The agents containing both selective serotonin and nor-adrenaline reuptake inhibitors (SSRIs and NRIs) showed potent swimming and climbing activity in FST as compared to those acting selectively on one particular system. Such chemicals are known to be more operative in treatment of resistant stressed patients [41, 42]. Based on these statements AHM and its active fraction AHE might considered to be beneficial by effecting both behavioral contributions and neurochemicals (SSRIs and NRIs) due to the presence of polyphenols [13–15]. Swamy and colleagues 2011, described that combination of polyphenols showed potent antidepressant activity by enhancing swimming period in FST in rats [43]. The possible difference in behavior among AHM and AHE with other fractions might be due to the occurrence of various bioactive potent phytochemicals.

Oxidative stress might be the underlying cause related to the pathophysiology of depression or associated behavioral changes [44]. The link of oxidative state with depression was analyzed by chronic mild stress (CMS) paradigm in repeated dose experiment and brain tissue oxidative status was analyzed after behavioral testing. The present findings supported that oxidative stress may be disordered in depressed rats, which is demonstrated by abnormal oxidative stress marker levels. In this investigation, we found in depressed rats: 1) the brain antioxidant levels were lower control (CMS) group; 2) the brain MDA, H_2_O_2_ and NO levels were higher in control (CMS) group. *A. hydaspica* AHM and AHE significantly ameliorates brain antioxidant levels and showed marked antidepressant and anxiolytic potential by decreasing oxidative stress. Brain tissue antioxidant state was analyzed because the brain consumes about 20% of the oxygen available through respiration but it has limited ability to counteract oxidative stress. Therefore, because of its high oxygen demand, the brain is the most susceptible organ to oxidative damage [11]. Stress increase free radicle generation which leads to oxidative stress (OS), OS has been implicated in various pathological states of the brain, including Alzheimer’s disease, Parkinson’s disease, Huntington’s disease, amyotrophic lateral sclerosis, ischaemic and haemorrhagic stroke [10]. Novel antioxidants may offer an effective and safe means of bolstering body’s defense against free radicals. Central nervous system defense system can be activated/modulated by exogenous antioxidants such as polyphenols, flavonids, terpenoids, fatty acids etc. There is ample scientific and empirical evidence supporting the use of antioxidants for the control or slowing down progression of neurodegenerative disorders [45]. Plant extracts treatment that possesses antioxidant capacity also potentiate antidepressant potentials in in vivo studies [46, 47]. Function of flavonoids with antidepressant and anxiolytic potential has been established in many plants in traditional [48]. The antioxidant potential of the AHM and AHE may complement their antidepressant and anxiolytic like actions [14]. Furthermore, current findings are reinforced by the study of Zu et al., 2012 reported that more ingestion of green tea outcomes in lower prevalence of depressive signs [49]. Presence of polyphenols e.g. gallic acid, catechin, rutin, caffeic acid, 7–*0*-galloyl catechin, catechin and methyl gallate [16, 40, 50], in *A. hydaspica* might be responsible for the observed mode boosting activity in FST, TST and EPMT. Previous studies also affirm that Polyphenols have antidepressant potential and reverse anxiety-related behavior of rodents without side effects [51, 52].

GC-MS analysis of *A. hydaspica* crude methanol extract (AHM) reveals the occurrence of various phyto-constituents. The chromatogram reveals that AHM is a complex mixture of various metabolites such as triterpenoids, fatty acids, vitamins, aromatic compounds etc. The therapeutic potential of identified compounds has been established from other plant species. Compounds possessing cytotoxic, antibacterial, antifungal, anti-inflammatory activities have been identified in AHM. 1, 2-Benzenedicarboxylic acid, mono (2-ethylhexyl) ester is major compound identified in AHM by GCMS, possess anticancer, anti-inflammatory and antioxidant potential [53]. Free radicle scavenging ability of this compound might be indirectly linked with antidepressant and anxiolytic potential of *A. hydaspica* by boosting brain antioxidant status. The occurrence of Vitamin E in the plant crude extract clues that plant is a source of effective antioxidant and is a toxicant protector, as Vitamin E is a well authenticated antioxidant, protective agent against drug induced toxicity. Besides, vitamin E is effective in various hematological disorders and other malignancies [54]. 2, 6-dimethyl-N-(2-methyl-à-phenylbenzyl) aniline is a derivative of aniline which has safer antimicrobial and in vitro antioxidant property [55]. Squalene, another constituent identified in GCMS is a natural triterpene, is known to have active oxygen scavenging activities. Presence of squalene in *A. hydaspica* might contribute to the observed anti-depressant potential by boosting brain antioxidants. It also possesses anticancer properties [56]. Phytol compounds occurrence in *A. hydaspica* indicates the relevance of anticancer, antimicrobial and anti-inflammatory prospective of plant [57]. α amyrin, a triterpene present in extract might also responsible for antidepressant and anxiolytic potential. Previous reports affirm that α amyrin decrease the immobility time in the behavior despair test in mice. The anxiolytic effects of the mixture of α/β-amyrin, isolated from the stem bark resin of Protium heptaphyllum, has been demonstrated by different animal models [58]. The results of that study showed that α/β-amyrin significantly decreased the number of crossings, grooming, rearing and the time of permanence and the number of entrances in the close arms whereas, increased the time of permanence and the number of entrances in the open arms [59]. The anti-anxiety and anti-depressant effect of *A. hydaspica* may be attributed to the presence of various compounds which might act in in additive manner, resulting in enhanced potency of active constituents.

## Conclusion

The present study provides the first evidence that *A. hydaspica* extract (AHM) and its derived fraction AHE produce antidepressant and anxiolytic effect by neutralization of ROS and improving brain antioxidant activity. This study provides a link of depressive and anxiety like behaviors with oxidative stress. The observed activities might be attributed to the occurrence of different phytochemicals. Finally, though the presence of receptors or transporters for polyphenols or other phytochemicals in brain tissues remains to be ascertained, compounds with multiple targets appear as a potential and promising class of therapeutics for the treatment of neurological disorders.

## Additional file

Additional file 1: (GCMS fragmentation pattern and compounds structure). (DOCX 1228 kb)
