# Supplementary material for: Anti-depressant and anxiolytic potential of Acacia hydaspica R. Parker aerial parts extract: Modulation of brain antioxidant enzyme status
Source: BMC Complement Altern Med. 2017 Apr 24;17:228. doi: 10.1186/s12906-017-1671-x (PMC5402641; doi:10.1186/s12906-017-1671-x)
Supplement: Additional file 1: — (GCMS fragmentation pattern and compounds structure). (DOCX 1228 kb) [file 12906_2017_1671_MOESM1_ESM.docx]

**Additional file**

**GCMS set up**


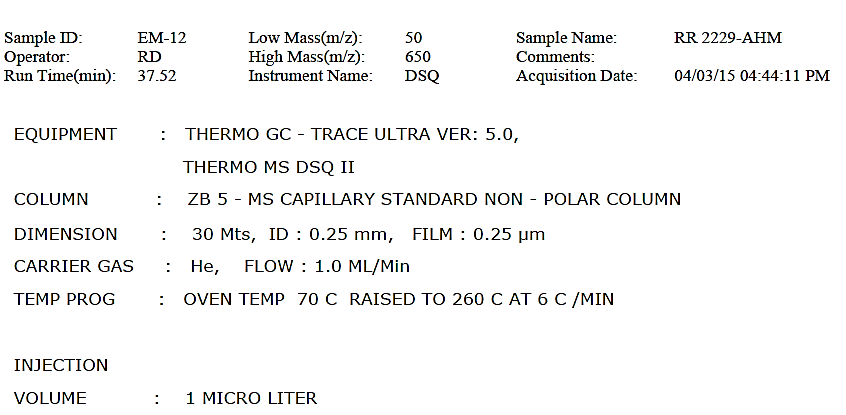


**GCMS data analysis**

Compounds peaks appear at different retention times were analyze on MS full scan mode. The fragmentation patterns of unknown compound in the original GC spectrum were compared with the compounds stored in computer library. The unknown compounds in GCMS chromatogram of *A. hydaspica* AHM extract were predicted by matching the MS fragmentation pattern of each compound peak with known compounds MS fragmentation pattern and probability. The compounds with highest probability were expected to be present in the plant extract.

**Comparison of spectrums through mass finders**

Only the compounds with peak area greater than 1% were shown. The minor peaks were excluded.

**Compound 1: RT 6.53**


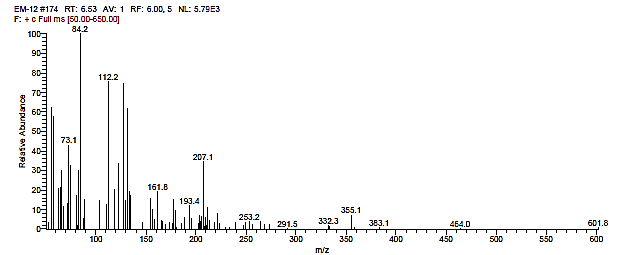


**Library Search ResultsLib. Search Graphic Table**


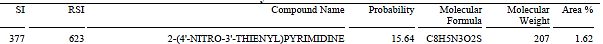


| **Compound structure Hit spectrum** | |
| --- | --- |
| C1:  Wiley9,  Entry# 157741, CAS#65868-26-0  Probability: 15.64  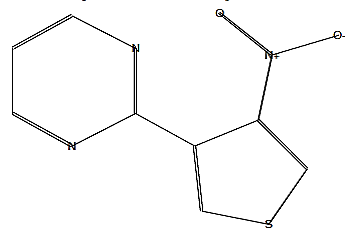  Pyrimidine, 2-(4-nitro-3-thienyl)- (CAS) | NL: 9.99E2  EM-12174 6.53 1  6.00, 5 5.79E3 + c Full  ms [50.00-650.00]  ****  NL: 9.99E2  SI 377, RSI 623, Wiley9,  Entry# 157741, CAS#65868-26-0,  2-(4'-NITRO-3'-  THIENYL) PYRIMIDINE |

**C2: RT 20.24 Library Search Results**


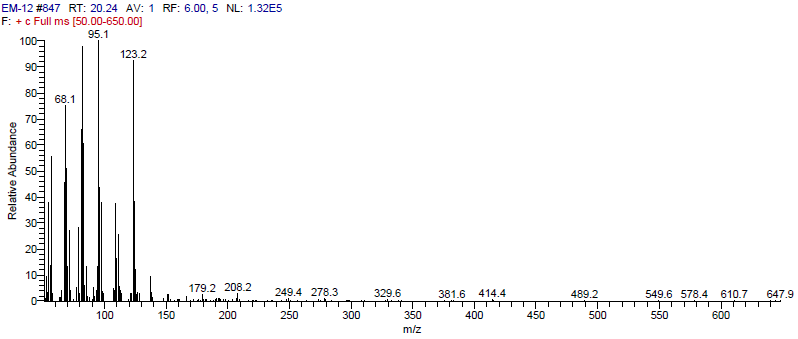

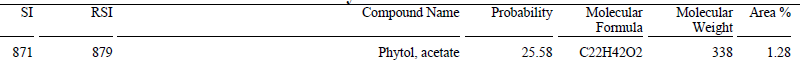


**Lib. Search Graphic Table**

| **Compound structure Hit Spectrum** | |
| --- | --- |
| C2:  mainlib, Entry#  7996, CAS# NA  Probability: 25.58    Phytol acetate | NL: 9.99E2  EM-12847 20.24  1 6.00, 5  1.32E5 + c Full  ms [50.00-650.00]    NL: 9.99E2  SI 871, RSI 879,  mainlib, Entry#7996, CAS# NA,  Phytol, acetate |

**C 3 : RT 25.52**


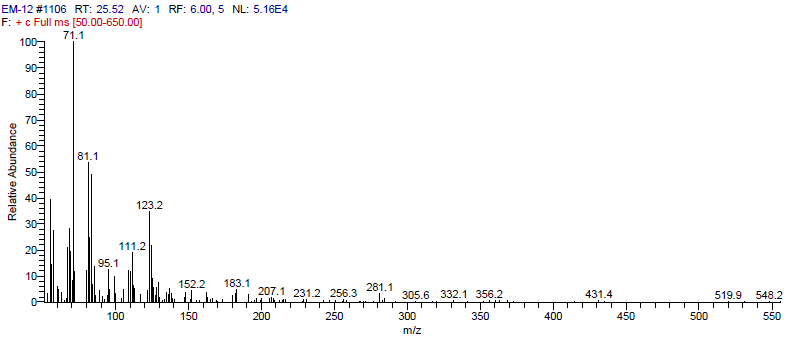


**Library Search Result**


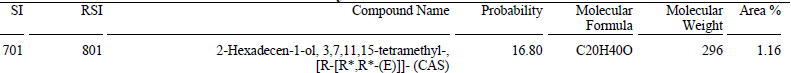


**Lib. Search Graphic Table**

| **Compound structure Hit Spectrum** |
| --- |

| C3:  Wiley9, Entry#  366710, CAS# 150-86-7  Probabibility:16.80    2-Hexadecen-1-ol, 3,7,11,15-tetramethyl-, [R-[R*,R*-€]]- (CAS), Phytol (CAS) | NL: 9.99E2  EM-121106 25.52 1 6.00, 5  5.16E4 + c Full ms  [50.00-650.00]    NL: 9.99E2  SI 701, RSI 801, Wiley9, Entry#  366710, CAS# 150-86-7,  2-Hexadecen-1-ol, 3,7,11,15-  tetramethyl-,[R-[R*,R*-(E)]]- (CAS) |
| --- | --- |

**C 4: RT 28.59Library Search Results**


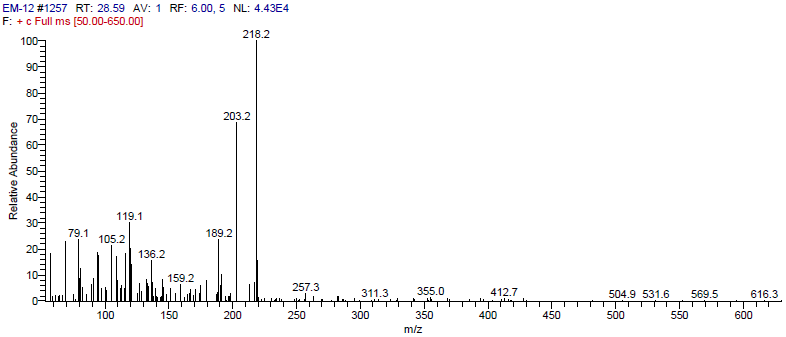

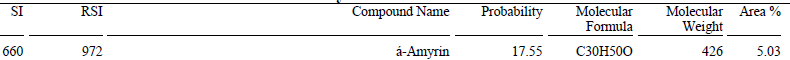


**Lib. Search Graphic Table**

| **Compound structure Hit Spectrum** | |
| --- | --- |
| C4:  Wiley9, Entry#  570119, CAS#  559-70-6, 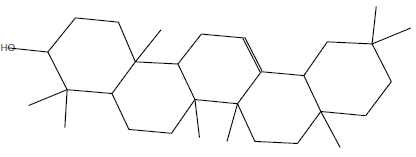 á-Amyrin  Olean-12-en-3-ol, (3á)- (CAS) | **NL: 9.99E2**  **EM-121257 28.59**  **1 6.00, 5**  **4.43E4 + c Full**  **ms [50.00-650.00]**    NL: 9.99E2  SI 660, RSI 972,  Wiley9, Entry#  570119, CAS#  559-70-6, á-Amyrin |

**C5: RT 30.51Library Search Results**


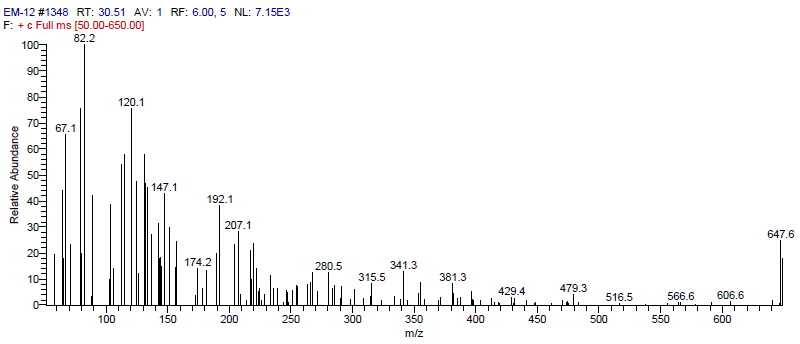

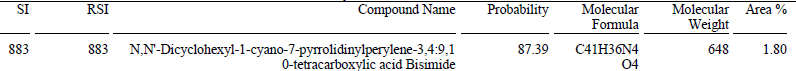


**Lib. Search Graphic Table**

| **Compound structure Hit Spectrum** | |
| --- | --- |
| C5:  Wiley9, Entry#  650496, CAS# NA  Probability: 87.39    N,N’-Dicyclohexyl-1-cyano-7-pyrrolidinylperylene-3,4:9,10-tetracarboxylic acid Bisimide | NL: 9.99E2  EM-121348 30.51 1 6.00, 5  7.15E3 + c Full ms [50.00-650.00    NL: 9.99E2  SI 883, RSI 883, Wiley9, Entry#  650496, CAS# NA,  N, N’-Dicyclohexyl-1-cyano-7-  Pyrrolidinyl perylene-3,4:9, 10-  tetracarboxylic acid Bisimide |

**C6: RT 31.66Library Search Results**


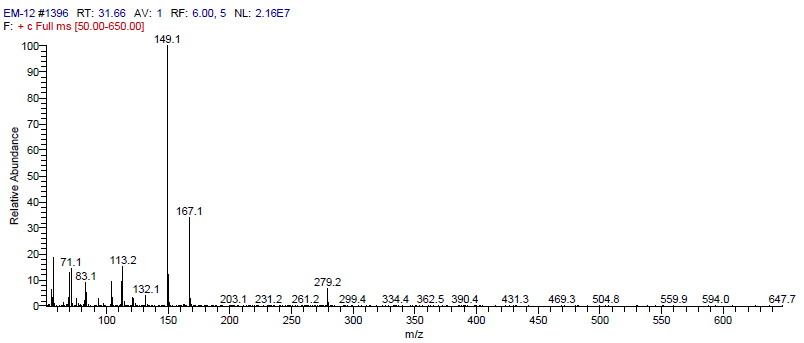

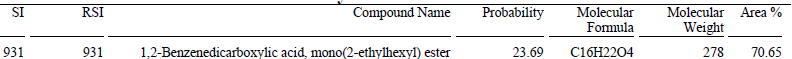


**Lib. Search Graphic Table**

| **Compound structure Hit Spectrum** | |
| --- | --- |
| C6:  Wiley9,  Entry# 324847, CAS#  4376-20-9  Probability:23.69    1,2-Benzenedicarboxylic acid, mono (2-ethylhexyl) ester, Mono(2-ethylhexyl) phthalate | NL: 9.99E2  EM-121396 31.66 1 6.00,  5 2.16E7 + c Full ms  [50.00-650.00]    NL: 9.99E2  SI 931, RSI 931, Wiley9,  Entry# 324847, CAS# 4376-20-9,  1,2-Benzenedicarboxylic acid,  mono(2-ethylhexyl) ester |

**C7: RT: 32.75Library Search Results**


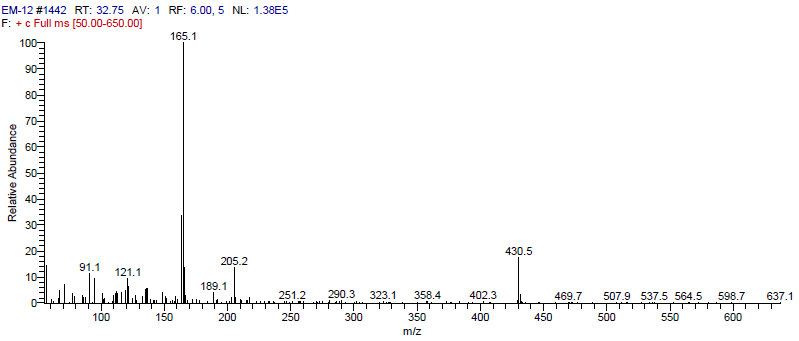

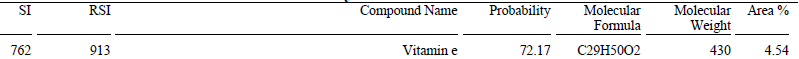


**Lib. Search Graphic Table**

| **Compound structure Hit Spectrum** | |
| --- | --- |
| C 7:  Wiley9, Entry#  574153, CAS#  59-02-9  Probability:72.17 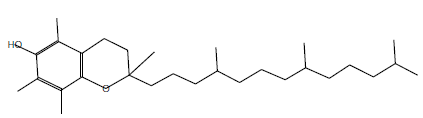 Vitamin E | NL: 9.99E2  EM-121442 32.75  1 6.00, 5  1.38E5 + c Full  ms [50.00-650.00]    NL: 9.99E2  SI 762, RSI 913,  Wiley9, Entry#574153, CAS#  59-02-9, Vitamin e |

**C8: RT 34.98Library Search Results**


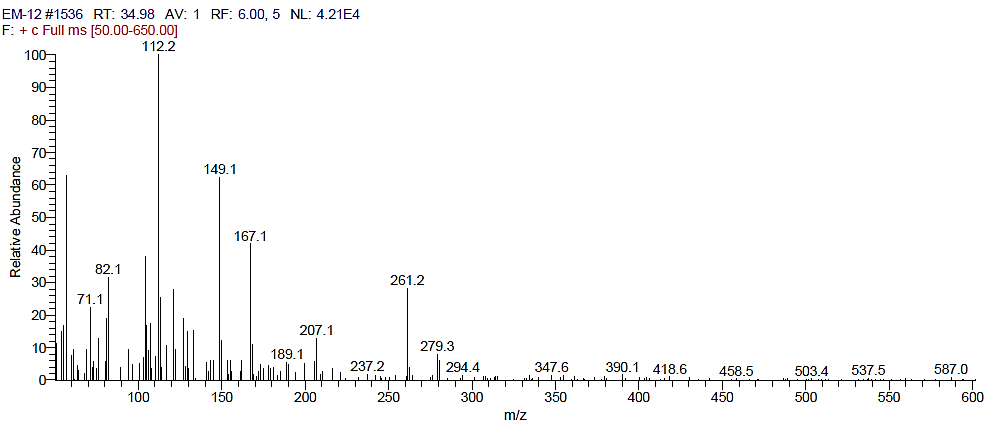

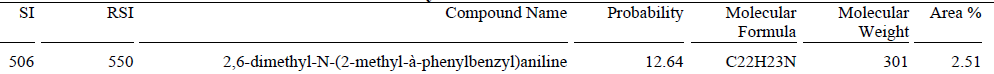


**Lib. Search Graphic Table**

| **Compound structure Hit Spectrum** | |
| --- | --- |
| C8:  Wiley9,  Entry# 377351, CAS#119971-00-5  Probability:12.64    2,6-dimethyl-N-(2-methyl-à-phenylbenzyl)aniline | NL: 9.99E2  EM-121536 34.98 1 6.00,5 4.21E4 + c Full ms  [50.00-650.00]    NL: 9.99E2  SI 506, RSI 550, Wiley9,  Entry# 377351, CAS#119971-00-5,  2,6-dimethyl-N-(2-methyl-àphenylbenzyl)  aniline |

**C9: RT 36.49Library Search Results**


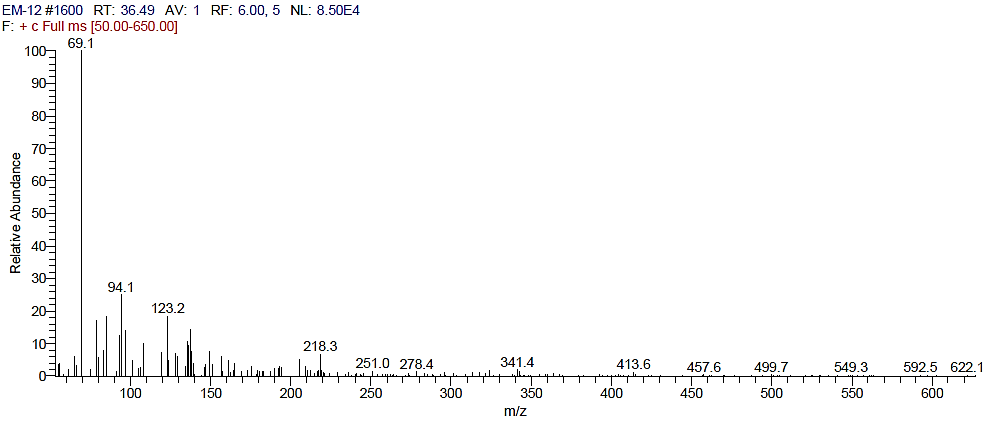

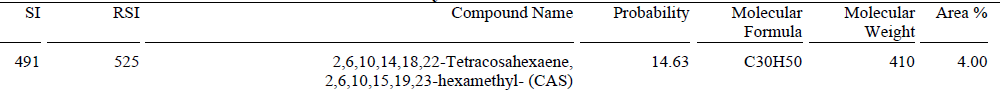


**Lib. Search Graphic Table**

| **Compound structure Hit Spectrum** | |
| --- | --- |
| C9:  Wiley9, Entry#554095,  CAS# 7683-64-9  Probability:14.63 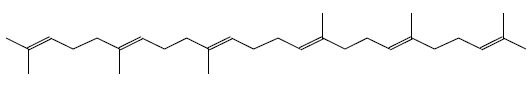 2,6,10,14,18,22-Tetracosahexaene, 2,6,10,15,19,23-hexamethyl- (CAS), Squalene | NL: 9.99E2  EM-121600 36.49 1 6.00, 5  8.50E4 + c Full ms  [50.00-650.00]    NL: 9.99E2  SI 491, RSI 525, Wiley9, Entry#  554095, CAS# 7683-64-9,  2,6,10,14,18,22-Tetracosahexaene,  2,6,10,15,19,23-hexamethyl- (CAS) |

**C10: RT 37.42Library Search resultLib. Search Graphic Table**


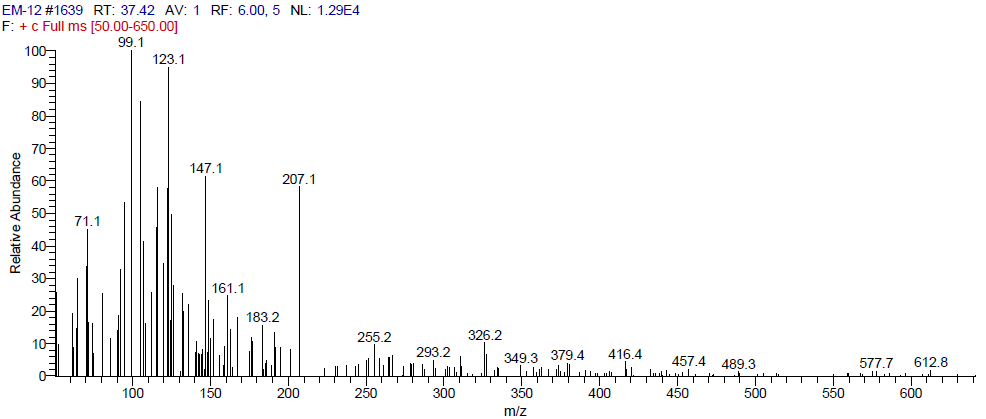

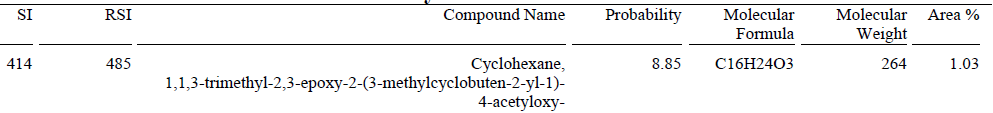


| **Compound structure Hit Spectrum** | | |
| --- | --- | --- |
| C10:  Wiley9, Entry#  292802, CAS# NA  Probability:8.85    Cyclohexane,1,1,3-trimethyl-2,3-epoxy-2(3- methylcyclobuten-2-yl)-4-acetyloxy- | NL: 9.99E2  EM-121639 37.42 1 6.00, 5  1.29E4 + c Full ms [50.00-650.00]    NL: 9.99E2  SI 414, RSI 485, Wiley9, Entry#  292802, CAS# NA, Cyclohexane,  1,1,3-trimethyl-2,3-epoxy-2-(3-  methylcyclobuten-2-yl-1)-4-acetyloxy |  |
